# Capillary electrophoresis for the assay of fixed-dose combination tablets of artesunate and amodiaquine

**DOI:** 10.1186/1475-2875-11-149

**Published:** 2012-05-03

**Authors:** N’Cho Christophe Amin, Marie-Dominique Blanchin, Michèle Aké, Jérôme Montels, Huguette Fabre

**Affiliations:** 1 Laboratoire de Chimie Analytique, Contrôle physico-chimique des médicaments, Institut des Biomolécules Max Mousseron, UMR 5247, Faculté de Pharmacie, Montpellier, BP 14491-34093, France; 2 Laboratoire de Chimie Analytique, Bromatologie, Chimie Minérale et Chimie Générale, Université de Cocody - UFR Sciences Pharmaceutiques et Biologiques, Abidjan, Côte d’Ivoire, BPV 34, West Africa

**Keywords:** Anti-malarials, Amodiaquine, Artesunate, Fixed-dose combination, MEKC

## Abstract

**Background:**

Quality control of drugs in formulations is still a major challenge in developing countries. For the quality control of artesunate and amodiaquine tablets in fixed-dose combination, only liquid chromatographic methods have been proposed in the literature. There are no capillary electrophoretic methods reported for the determination of these active substances, although this technique presents several advantages over liquid chromatography (long lifetime, low price of the capillary, low volumes of electrolyte consumption) in addition to simplicity. In this paper, a reliable capillary electrophoresis method has been developed and validated for the quality control of these drugs in commercial fixed-dose combination tablets.

**Methods:**

Artesunate and amodiaquine hydrochloride in bilayer tablets were determined by micellar electrokinetic capillary chromatography (MEKC). Analytes were extracted from tablets by sonication with a solvent mixture phosphate buffer pH 7.0-acetonitrile containing benzoic acid as internal standard. Separation was carried out on Beckman capillary electrophoresis system equipped with fused silica capillary, 30 cm long (20 cm to detector) × 50 μm internal diameter, using a 25 mM borate buffer pH 9.2 containing 30 mM sodium dodecyl sulfate as background electrolyte, a 500 V cm^−1^ electric field and a detection wavelength of 214 nm.

**Results:**

Artesunate, amodiaquine and benzoic acid were separated in 6 min. The method was found to be reliable with respect to specificity,linearity of the calibration line (r^2^ > 0.995), recovery from synthetic tablets (in the range 98–102%), repeatability (RSD 2–3%, n = 7 analytical procedures). Application to four batches of commercial formulations with different dosages gave content in good agreement with the declared content.

**Conclusion:**

The MEKC method proposed is reliable for the determination of artesunate and amodiaquine hydrochloride in fixed-dose combination tablets. The method is well-suited for drug quality control and detection of counterfeit or substandard medicines.

## Background

**Figure 1:**
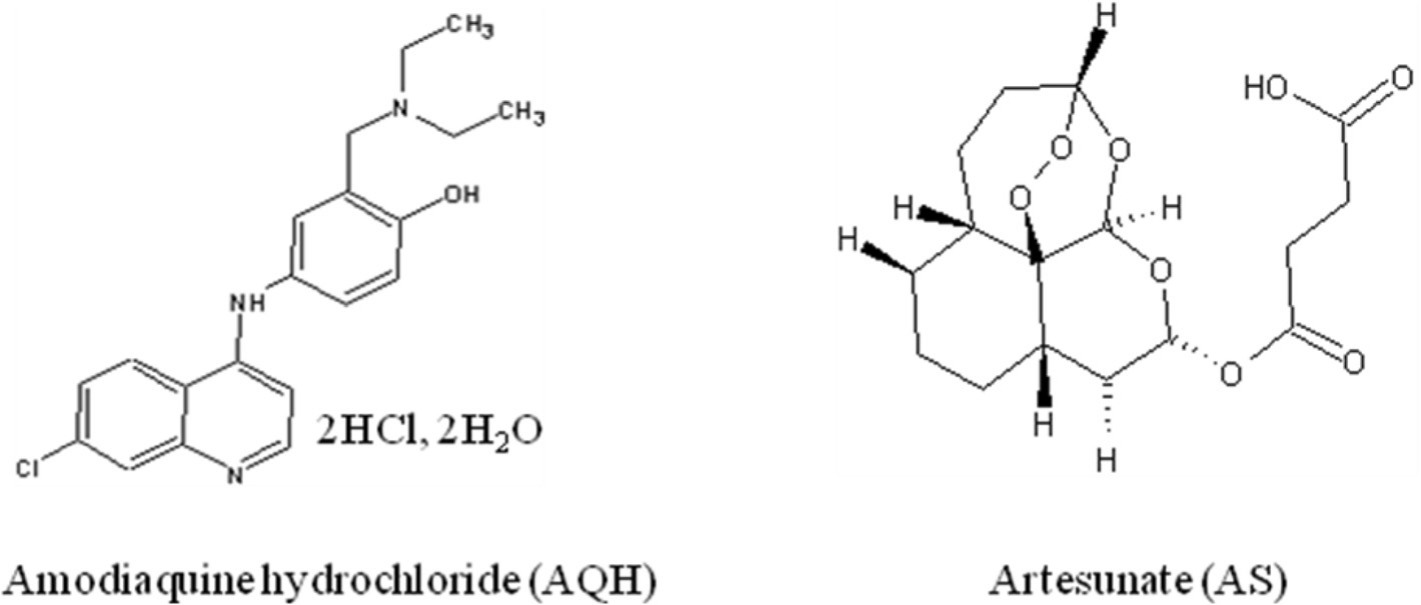
Chemical structure of amodiaquine hydrochloride (AQH) and artesunate (AS).

Malaria is the most important parasitic disease in the world which afflicts more than 800 million people. World Health Organization (WHO) recommends that artemisinin-based combination therapy (ACT) be used to counter the threat of *Plasmodium falciparum* resistance to artemisinin monotherapies and improve treatment outcome. Artesunate (AS) plus amodiaquine (AQ) (Figure 1) is one of the three WHO-recommended forms of ACT in Africa. Fixed-dose combination (FDC) formulations are strongly preferred and recommended over blistered co-packaged or loose tablets combinations to promote adherence to treatment [1]. FDC for artesunate (AS) and amodiaquine (AQ) was first registered in 2007 under the brand name ASAQ® (Winthrop) for public market and Coarsucam® (Sanofi) for private market. It is formulated as bilayer tablets to limit the physical contact between the active substances and avoid the degradation of AS which is accelerated in the presence of AQ [2]. Simultaneous determination of the two active substances appears rather difficult for several reasons such as the absence of chromophore for AS, the content difference between AS and AQ (weight ratio AS/AQ 1/2.7 in the formulations), the complexity of the formulation and the rapid degradation of AS in solution. Only two high performance liquid chromatographic (HPLC) methods using reverse mode separation have been reported for AS and AQ determination in FDC tablets [3,4]. In the method reported by Phadke *et al.*[3], both compounds are assayed in a single run at two different wavelengths (210 nm for AS, 300 nm for AQ) using a diode array detector. The run time was 9 min. In the paper of Gandhi *et al.*[4], internal standardization with artemether is used to determine the two compounds at a unique wavelength (220 nm) using two test solutions. AS is determined on a mixed concentrated test solution of tablet (500 mg L^-1^ AS; 1.5 g L^-1^ AQ hydrochloride (AQH)) and AQH is determined on a dilution 1/10 of this test solution. The run time is about 16 min.

No capillary electrophoresis (CE) method has been reported concerning the determination of AS and AQ in pharmaceutical formulations. CE has been used for the separation of AS from its hydrolysis products [5], from artelinic acid, a potential anti-malarial drug [6] and for AS determination in caco-2 cells [7]. AQ has been used in CE as a model compound to test the efficiency of a coating agent to prevent adsorption of basic substances on the capillary wall [8]. Since CE presents distinct advantages over HPLC in terms of reduced operating costs and simplicity, the possibility of using CE for the quality control of AS and AQ in fixed-dose combined formulations has been investigated in this paper.

## Methods

### Chemicals

All chemicals and solvents were of analytical grade. Deionized water distilled from a quartz apparatus was used throughout. AS and AQH were from Maphar (Casablanca, Morocco). Benzoic acid (internal standard) was from Merck (Darmstadt, Germany). Sodium dihydrogen phosphate was from Prolabo (Fontenay-sous-Bois, France), sodium dodecyl sulfate (SDS) was from Fluka (Neu Ulm, Germany). HPLC grade acetonitrile and sodium hydroxide were from Carlo Erba (Val de Reuil, France), hydrochloric acid was from Sigma-Aldrich (Steinheim, Germany). Coarsucam® (Sanofi-Aventis, Morocco) and ASAQ Denk® (Denk Pharma, Germany) commercial tablet formulations were purchased in Côte d’Ivoire.

### Solutions

The background electrolyte solution was a 25 mM sodium borate buffer (pH 9.2) solution containing 30 mM SDS. The internal standard solution (ISS) was a 50 mg L^-1^ of benzoic acid (BA) solution in 10 mM phosphate pH 7 – acetonitrile (60: 40, v/v) solvent mixture.

A concentrated standard solution (2 g L^-1^ AS and 7 g L^-1^ AQH) was prepared by sonication of about 20 mg AS and 70 mg AQH (accurately weighed) in 10 mL of ISS (= standard solution solution AS) for AS determination. This solution was diluted (1/100, v/v) in the ISS (= standard solution AQ) for AQH determination.

Five tablets were weighed accurately and crushed with a pestle in a porcelain mortar and thoroughly homogenized. A quantity of tablet powder equivalent to about 20 mg of AS and 70 mg of AQH was accurately weighed in a 10 mL volumetric flask. After addition of about 7 mL of ISS, the flask was subjected to ultrasonication for 10 min with intermittent shaking, then ISS was added to the mark. The suspension was centrifuged at 5,000 rpm for 5 min. and the supernatant (= test solution AS) corresponding to a theoretical concentration of 2 g L^−1^ AS (and 7 g L^-1^ AQH) was used for AS determination. This solution diluted (1/100, v/v) in the ISS for AQH determination (= test solution AQ) had a 0.07 g L^-1^ theoretical AQH concentration). Standard and test solutions were stable for at least 24 h at ambient temperature.

### Apparatus and operating conditions

A Beckman P/ACE MDQ (Fullerton, CA) instrument equipped with a photodiode array detector was used. Separation was carried out on a fused-silica capillary, 30 cm long (20 cm to the detector), 50 μm internal diameter (TSP, Composite Metal Services, Hallow, Worcs, UK), housed in a cartridge with a 200 μm × 800 μm detection window. Prior to its first use, the capillary was preconditioned by washing at 20 psi for 20 min with a 0.1 M sodium hydroxide solution, and then flushed with water for 5 min. Every working day a preconditioning was carried out with 1 M hydrochloride acid followed by 1 M sodium hydroxide, water, and electrolyte buffer at 20 psi for 5 min.

The different stages of the proposed method for AS and AQ determination are given in Table 1. Standard and test solutions were placed in bracketting sequence and duplicate injections were used in each case. Average corrected peak areas (peak areas divided by their respective migration times) of analyte/IS were used for calculations.

**Table 1:** Operating conditions

**Operations**	
1. Capillary rinse	1 M HCl; 2 min; 20 psi
2. Capillary rinse	1 M NaOH; 1 min; 20 psi
3. Capillary rinse	Electrolyte solution; 1 min; 20 psi
4. Sample introduction (anodic side)	Analyte; 3 secondes; 0.3 psi (3.5 nL)
5. Wait	Water; 0 seconde
6. Separation	6 min; 10 kV (500 V.cm-1); 0.17 min ramp voltage; 25°C (i = 50 μA)
7. Detection	UV; 214 nm; spectral bandwith 10 nm; acquisition rate 4 Hz; filter normal
8. Autozero	1 min

## Results and discussion

To achieve the separation in the shortest time possible, all experiments were carried out on a short capillary (30 cm long, 20 cm effective length) with an internal diameter of 50 μm for an efficient dissipation of heat produced by Joule effect.

### Preliminary studies in capillary zone electrophoresis

First of all, the possibility of using capillary zone electrophoresis (CZE) was investigated since the pKa values of AS (pKa 4.3 (−)) and AQH (pKas 7.1 (+) and 8.1 (+)) show that they can be separated as anion (AS) and cation (AQ). Background electrolyte solutions in the pH range 7–8 were investigated since at these pH values, AS and AQ are under anionic and cationic forms respectively, and there is a high electro-osmotic flow (EOF) for a fast separation. Using 100 mM phosphate pH 7 and applying a 10 kV separation voltage, satisfactory separation was obtained with a standard solution (2.0 g L^-1^ AS, 0.07 g L^-1^), AQ being eluted first as cation (migration time 2.73 min) and AS as anion at 8.23 min. after the EOF (3.93 min.). The high concentration of AS (2.0 g L^-1^) was needed to have the required sensitivity for AS determination. Test solutions from tablets contain 2.0 g L^-1^AS and 7 g L^-1^ AQH. At this high concentration, AQ disturbed strongly the baseline for an accurate determination of AS, preventing CZE to be used for AS determination in FDCs. Further investigations were carried out in Micellar Electrokinetic Capillary Chromatography (MEKC).

### Investigations in micellar electrokinetic capillary chromatography

The different steps of optimization in MEKC are described below.

#### Optimization of the surfactant concentration

Sodium dodecyl sulfate (SDS) was selected as micellar additive to the background electrolyte as it is the most used anionic surfactant in MEKC and has a low UV absorbance. A 25 mM sodium borate buffer (natural pH 9.2) was chosen for its high buffer capacity (pKa 9.2) and its low UV absorbance. At this pH, a high EOF is provided which speeds up the separation.

**Figure 2:**
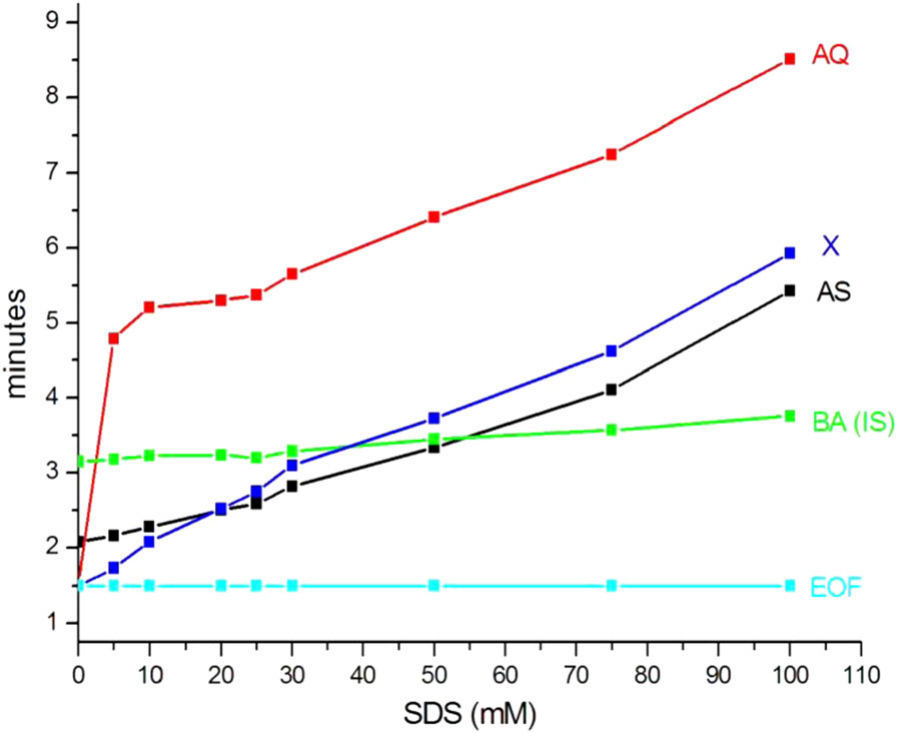
**Influence of SDS concentration on MTs of AS, AQ, IS and X (AQ impurity).** Operating conditions: 25 mM sodium tetraborate buffer pH 9.2 with SDS 0 – 100 mM; Capillary, 50 μm I.D. × 30 cm; 15 kV; 25°C °; λ = 214 nm; inj 3,5 nL.

Figure 2 shows the influence of SDS concentration (0–100 mM) on the migration times (MTs) of AS and AQ. At pH 9.2, AS which has a full negative charge, partitions between the micelles and the background electrolyte both through hydrophobic and electrostatic interactions. Because it is repelled from the micelles, it is eluted first. AQ, which is uncharged (eluted in the EOF at 0% SDS concentration), partitions through hydrophobic interactions and is eluted late. Selectivity was adjusted by varying the SDS concentration. Increasing SDS concentration has little influence on EOF velocity (a slight decrease is observed due to compression of the double layer), but dramatically influences the solute mobilities. As the number of micelles is increased, the concentration of solute in the micelles increases resulting in a lower mobility and change in selectivity. Resolution was satisfactory at 5, 10, 30, 75 and 100 mM SDS concentration. The best peak shape and peak efficiency was obtained at a 30 mM SDS concentration which was selected for further experiments as the best compromise in terms of resolution of AS from an AQ impurity, peak shape, analysis time and reasonable current (50 μA). No further optimization was carried out as function of voltage and borate concentration buffer.

#### Selection of a sample solvent

It appeared very difficult to find a suitable solvent to extract quantitatively both AQH and AS from tablets. Water in which AQH is very soluble could not be used as AS is not soluble in water at the concentration needed for its determination (2 g L^-1^). Methanol, and methanol–water mixture (1: 1; v/v) which could be potential extraction solvents [9] led frequent current breakdowns due to bubble formation during the separation. The next approach was to use a 10 mM phosphate buffer pH 3- acetonitrile mixture (60: 40, v/v) which has been successfully used for AS and AQH extraction from Coarsucam® tablets analysed in HPLC (Afssaps, personal communication). No current breakdown occurred and injections were repeatable. The stability of AS in this solvent was assessed.

**Figure 3:**
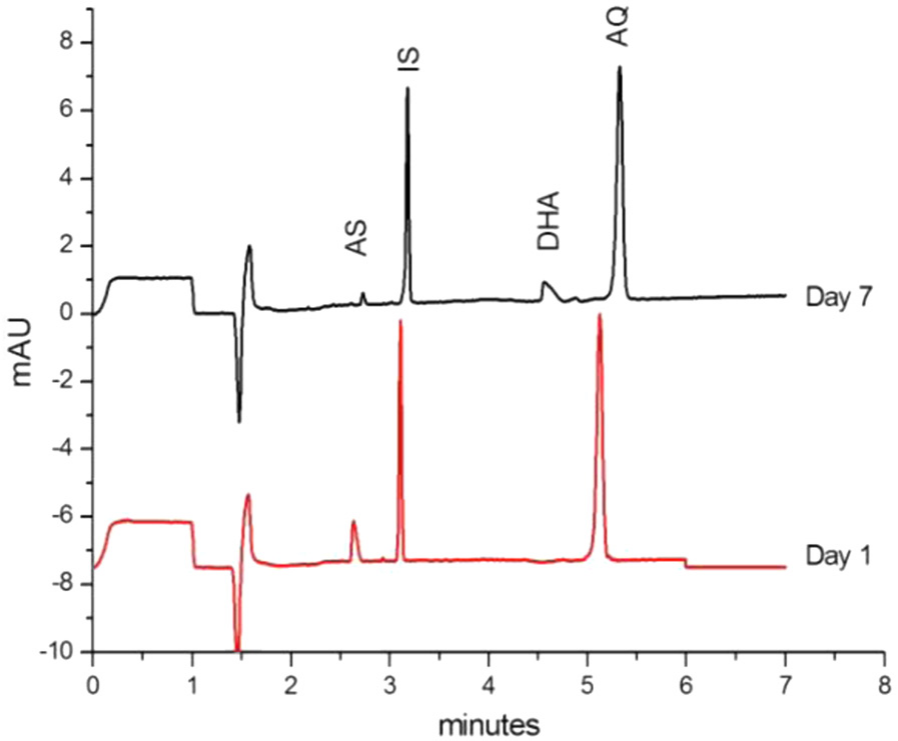
**Stability of a standard solution. AS 2 g L**^**-1**^**, AQH 70 mg L**^**-1**^**, and IS 70 mg L**^**-1**^**in acetonitrile – 10 mM phosphate pH 3 (40:60, v:v) stored at room temperature.** Operating conditions: background electrolyte, borate – SDS (25 mM sodium tetraborate buffer pH 9.2 – 30 mM SDS); capillary, 50 μm I.D. × 30 cm (20 cm); inj 3.5 nL; 15 kV; 25 ◦C; detection, λ = 214 nm. Dihydroartemisinin (DHA), a degradation product of AS was observed.

Electropherograms presented in Figure 3 show that the degradation of a standard solution of AS and AQ in this solvent mixture gives rise after 7 day storage at ambient temperature, to a compound eluted at a MT of about 4.5 min., which is assumed to be the hydrolysis product of AS, dihydroartemisinin (neutral compound) [5,10-12].The stability study of AS in the same solvent mixture carried out over a 24 h period showed that AS in test and standard solutions is stable only 4–5 h at ambient temperature. Since hydrolysis is favoured in acidic medium, the pH of the buffer was changed from 3.0 to 7.0 to improve the stability. Hence, the sample solvent used in the proposed method is a solution of internal standard in a 10 mM phosphate buffer pH 7.0- acetonitrile mixture (60: 40, v/v) which provides quantitative extraction of both compounds. These solutions are stable at least 24 h at ambient temperature.

#### Optimization of the injected volume and sample concentration

It is well known that injection of samples previously dissolved in an organic solvent affects peak efficiency and shape in MEKC, especially if the injected volume is increased as it causes the micelles near the sample zone to collapse [13]. Since acetonitrile (40%, v/v) was needed to solubilize the analytes from the tablets, the injection volume was reduced to about 3.5 nL (0.6% of the capillary volume) in order to maintain satisfactory peak efficiency and peak shape. Injection of larger volumes or more concentrated solutions (> 1 g L^-1^ AQH) resulted in peak splitting.

#### Selection of an internal standard

The use of an IS is needed for quantitative analysis in CE to take into account small variations of the injected volumes due to the injection system. In addition, if it is added in the extraction solvent (test solution) or dissolution solvent (standard solution) it takes also into account possible variations due to solvent evaporation. Among potential IS candidates, procaine (pKa 9), phenobarbital (pKa 7.4), and benzoic acid (pKa 4.3) were tested. Benzoic acid was found to be suitable as it is eluted between AS and AQ, is separated from AQ impurity (fig 3), gives a peak of acceptable efficiency and yields repeatable extractions of AS and AQ from tablets.

#### Selection of detection wavelength

Due to the lack of chromophore in AS, detection at a low wavelength was needed for AS determination. A 214 nm wavelength gave the best signal-to-noise ratio for AS. This allows the use of a UV detector with filter.

#### Capillary rinse between injections

Various rinse solutions (sodium hydroxide 0.1 M and 1 M; hydrochloric acid 0.1 M and 1 M) and rinsing time (1–3 min) were tested to avoid capillary fouling. Best performances were obtained using rinse cycles indicated in Table 1.

**Figure 4:**
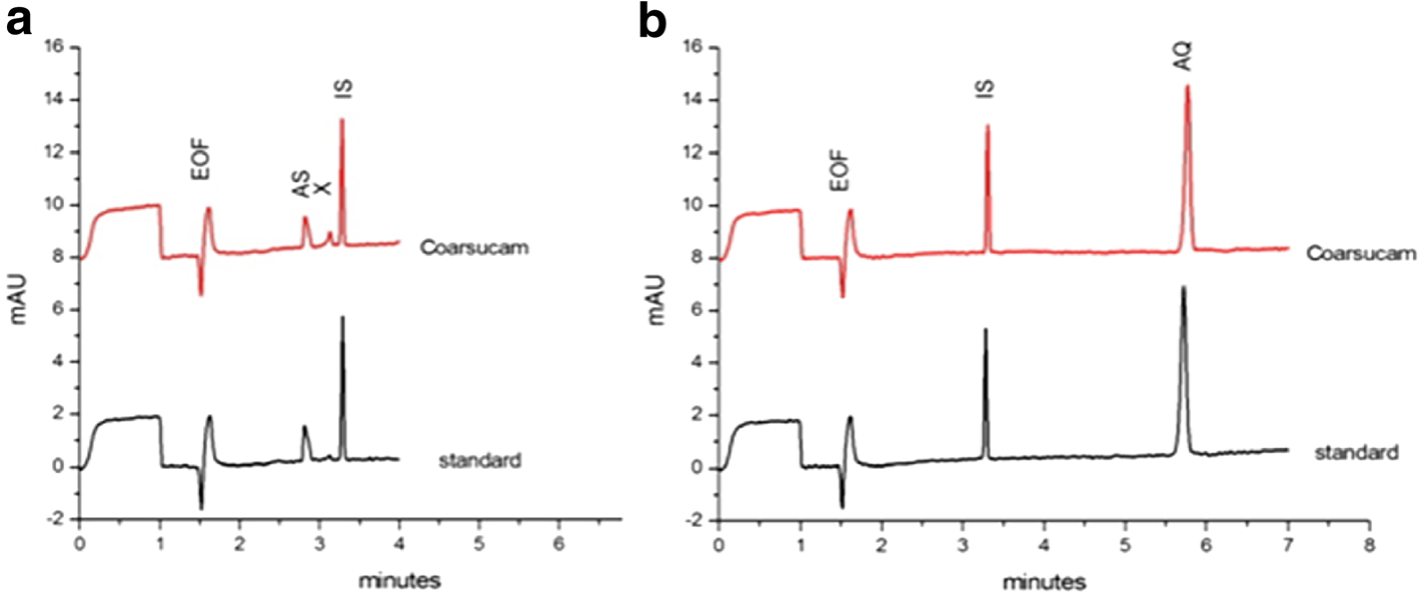
**Determination of AS, 2 g L**^**−1**^**(a) and AQH, 70 mg L**^**−1**^**(b).** Electropherograms of a standard solution (lower trace) and Coarsucam® test solution (upper trace). Conditions as in Figure 3.

Typical electropherograms of standard and test solutions analysed under the final selected conditions of Table 1 are shown in Figure 4. The MTs of AS, BA and AQ are respectively 2.82, 3.29, and 5.65 min. The small peak eluted at 3.13 min. is an impurity of AQ, which is evidenced when AQH is present at high concentration.

### Evaluation of method performances

The method was validated according the guidelines of the international conference of harmonisation [14]

#### Selectivity

**Figure 5:**
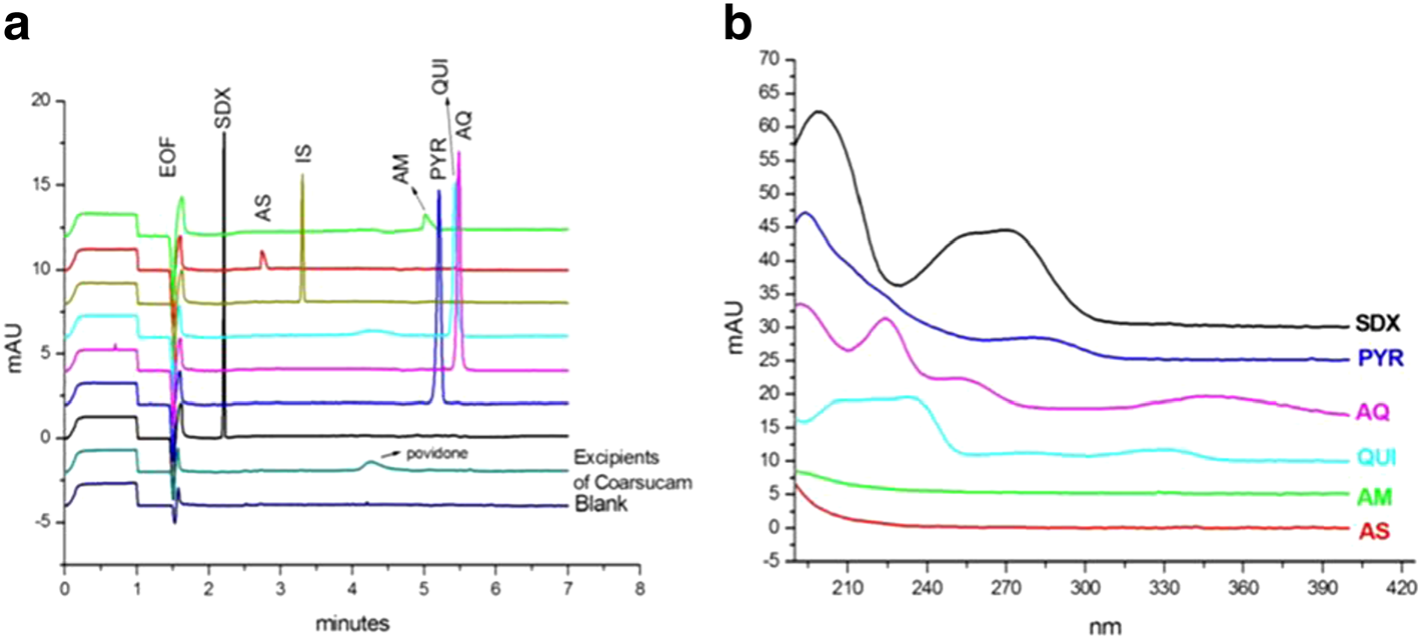
**(a) Selectivity towards formulation excipients and common anti-malarials; (b) UV spectra of anti-malarials recorded with the diode array detector.** Experimental conditions as in Figure 3.

The non-interference of ingredients present in the tablet formulations (Table 2) on the analyte peaks was assessed (Figure 5a) by injecting a blank solution of excipients. No interference was noted. As it was also considered that the method could be potentially applicable to detect counterfeit anti-malarial formulations [15], the selectivity was also tested (Figure 5a) towards common anti-malarials: artemether (AM), lumefantrine (LUM), quinine (QUI), pyrimethamine (PYR), and sulphadoxine (SDX).SDX, AM, LUM, PYR are separated from AS and AQ. QUI which is eluted at a MT very close to AQ could be differenciated from AQ by its UV spectrum (Figure 5b). Lumefantrine, which is a very hydrophobic compound, was not soluble in the dissolving solvent.

**Table 2:** Excipients of the different pharmaceutical formulations analysed

**Excipients**	**Coarsucam**®	**ASAQ Denk**®
Sodium croscarmellose	x	x
Povidone K30	x	
Magnesium stearate	x	x
Colloidal anhydrous silica	x	x
Calcium carbonate	x	
Maize starch	x	
Microcrystalline cellulose	x	x
Talc		x
Calcium hydrogen phosphate dihydrate		x
Lactose monohydrate		x

#### Linearity of the response function

The linearity of the response function (corrected peak areas, CPA analyte/IS) *vs* analyte concentration was assessed on standard solutions over the range 1–3 g L^-1^ for AS and 35–105 mg L^-1^ for AQH at 5 concentration levels corresponding to 50, 75, 100, 125, and 150% of the target concentration of AS (2 g L^-1^) and AQH (70 mg L^-1^).

Regression equations calculated using the least-squares method were:

(1) $$\text{CPA }\left(\text{AS}/\text{IS}\right)=\left(0.000174\pm 0.000013\right)\text{AS mg}\;{\text{L}}^{-1}–\left(0.0216\pm 0.0260\right);{\text{ r}}^{2}=0.999$$

(2) $$\text{CPA }\left(\text{AQH}/\text{IS}\right)=\left(0.0156\pm 0.0009\right)\text{ AQH mg}\;{\text{L}}^{-1}+\left(0.059\pm 0.063\right);{\text{ r}}^{2}=0.996$$

with the confidence intervals calculated at α = 0.05, analysis of variance shows that the relationship is linear and that the regression line passes through the origin.

#### System precision

The precision of the system was assessed by injecting seven times successively a standard solution at the target concentration and a test solution. The RSD values were typically better than 0.2% for RMTs (MTs relative to IS) and in the range 1 – 3% for RCPAs (CPAs relative to IS). Similar RMTs (0.85 for AS and 1.70 for AQ) were obtained on a same day, on different days, on different capillaries and different instruments. RMTs can be used together with UV spectra to confirm the identity of the drugs.

#### Accuracy: Recovery studies

The accuracy of the method was assessed by performing recovery experiments. Three series of independent determinations were carried out, each time at three concentration levels of AS and AQH. For each series, analytical placebos spiked with amounts of AS and AQH corresponding to 80, 100 and 120% of the target concentration were prepared. Recoveries of AS and AQH were calculated against a standard solution at the target concentration prepared in duplicate (Table 3). AS and AQH mean recoveries were at 101-102% and 98-99% respectively.

**Table 3:** Recovery studies for AS and AQ

**Exp. No.**	**Theoretical content (%)**	**Added amount (mg)**	**Found amount (%)**	**Recovery (%)**	**Mean recovery ± s (%)**
AS					
1		15.84	16.23	102	
2	80	15.99	16.12	101	101 ± 0.8
3		16.16	16.35	101	
1		19.85	20.21	102	
2	100	19.97	20.61	103	102 ± 0.7
3		20.06	20.48	102	
1		23.57	23.46	100	
2	120	24.00	24.54	102	101 ± 1.4
3		24.17	24.44	101	
AQH					
1		55.26	54.74	99	
2	80	55.65	55.03	99	99 ± 0.3
3		56.81	56.47	99	
1		69.3	68.49	99	
2	100	70.31	69.16	98	98 ± 0.9
3		70.87	68.84	97	
1		83.2	82.69	99	
2	120	85.05	83.06	98	99 ± 1.1
3		93.92	93.47	100	

#### Limit of quantification (LOQ)

The LOQ (signal-to-noise ratio of 10) is about 330 mg L^-1^ for AS and 3.5 mg L^-1^ for AQH. These limits correspond to LOQs of 16.5 mg and 0.2 mg of AS and AQH respectively in a tablet of 700 mg.

### Application to commercial tablet formulations

Different fixed-dose combination tablets (Coarsucam® and ASAQ Denk®) were analysed for AS and AQH content using the proposed CE method. Duplicate independent determinations were carried out except for Coarsucam® 50 mg AS – 176.32 mg AQH for which seven replicate independent determinations were carried out to test the repeatability of the entire analytical procedure (Table 4). The repeatability expressed as the relative standard deviation was, 2.04% for AS and 3.00% for AQ.

**Table 4:** Assay of commercial tablet formulations

**Tablets**	**% of the labelled claim**	
	**AS**	**AQH**
Coarsucam®; 25 mg AS – 88.16 mg AQH (67.5 mg AQ base)	101.6	99.6
Coarsucam®; 50 mg AS – 176.32 mg AQH (135 mg AQ base)	98.1	100.2
Coarsucam®; 100 mg AS – 352.64 mg AQH (270 mg AQ base)	97.0	98.1
ASAQ Denk®; 100 mg AS – 352.64 mg AQH (270 mg AQ base)	98.0	99.5

## Conclusion

MEKC can be used for the determination of AS and AQH in fixed-dose combination tablet formulations. Satisfactory results were obtained for method validation according to the ICH guidelines with respect to selectivity, linearity of the calibration line, accuracy and precision. The method presents an interesting alternative to liquid chromatography for drug quality control and detection of counterfeit or substandard medicines in developing countries. It should be emphasized that, thanks to a European project, low-cost capillary electrophoresis machines are now installed in Mali, Cambodia and will be soon installed in Congo and Senegal for this aim [16]. The main advantages of CE over liquid chromatography are the low running cost: low price of the capillary (about 4 dollars) which has a long lifetime (more than 300 injections) and low consumption of separation electrolyte (in the order of 10 mL per day) which makes the price of a test more cost efficient.

## Abbreviations

ACT = artemisinin-based combination therapy; Afssaps = Agence française de sécurité sanitaire des produits de santé; AM = Artemether; AQ = Amodiaquine; AQH = Amodiaquine hydrochloride; AS = Artesunate; BA = Benzoic acid; CE = Capillary electrophoresis; CPA = Corrected peak areas; CZE = Capillary zone electrophoresis; EOF = electro-osmotic flow; FDC = Fixed-dose combination; ISS = internal standard solution; LUM = Lumefantrine; MEKC = Micellar Electrokinetic Capillary Chromatography; MT = Migration time; PYR = Pyrimethamine; QUI = Quinine; RCPA = Relative CPA; RMT = Relative MT; SDS = Sodium dodecyl sulfate; SDX = Sulphadoxine; WHO = World Health Organization.

## Competing interests

The authors declare that they have no competing interests.

## Authors’ contributions

NCA: study design, sample preparation, data collection, analysis and interpretation of data, drafting of manuscript. M-DB and MA: study design and manuscript preparation. JM: technical contribution. HF: conception of the study, supervision on the progress of the study and revision of the manuscript. All authors read and approved the final manuscript.
